# The Interplay of Pathological Gambling, Substance Abuse, and Financial Ruin: A Case Report

**DOI:** 10.7759/cureus.69684

**Published:** 2024-09-18

**Authors:** Selvameenatchi R, Sivabackiya C, Ramya Rachel Jetty, Jane Rinita John De Britto, Arul Saravanan R

**Affiliations:** 1 Psychiatry, Sri Ramaswamy Memorial (SRM) Medical College Hospital and Research Institute, SRM Institute of Science and Technology, Chennai, IND

**Keywords:** financial debts, gambling disorder, online gambling, pathological gambling, substance use

## Abstract

Gambling, which was previously restricted to physical casinos and card rooms, has become readily accessible through internet platforms and virtual card games as a result of recently developed technological advances. The increase in the prevalence of pathological gambling (PG) disorder has been attributed to the extensive availability of online gambling platforms and the covert advertising that is associated with them. In this case, we examine an individual with a PG disorder who is intrigued by online gambling. This individual also has a co-occurring drug use disorder and is burdened by significant financial debt and family problems. These issues have arisen due to an uncontrollable impulse to gamble in online games. The participants initially achieved transient success in these online games, which led to a strong desire to always participate in them, neglecting their personal and professional responsibilities and overall well-being. After depleting the individual's financial savings entirely, he turned to stealing from his own home in order to continue gambling and recover their losses. This has been facilitated by the ease and convenience of online money transactions through websites and mobile applications, which has resulted in a greater engagement and understanding of online gambling.

## Introduction

Gambling disorder is officially acknowledged in the Diagnostic and Statistical Manual of Mental Disorders-5 (DSM-5) as a psychiatric illness that falls under the nonsubstance-related category [1]. Gambling disorder is a psychiatric condition that impacts around 0.4-2% of the world's population [2]. Pathological gambling (PG) is characterized by a strong need to gamble, a lack of control over gambling activity, and a persistent and harmful pattern of frequent gambling despite experiencing negative effects [3]. Some may view PG as mere entertainment or a way to relieve stress, but for many individuals, it becomes a chronic condition that manifests as a consistent and uncontrollable gambling behavior. Problem gambling has a profound impact on a person's daily functioning and produces considerable psychological suffering. It is marked by continuous gambling, escalating financial debt, challenges in family and social relationships, occupation difficulties, and legal troubles, which may end up in comorbid illnesses like substance use, anxiety, depression, and thoughts of suicide [4]. A systemic review and a meta-analysis discovered that 2.43% of the adult population was at risk for gambling, while 1.29% revealed problem gambling or PG [5]. The existence of the condition was not easily noticeable to the general public or healthcare experts during that period, resulting in it being frequently undiagnosed [6]. This case report presents a 30-year-old married male with alcohol dependence and recent onset PG disorder. The patient's extensive participation in online rummy games had resulted in a substantial amount of debt, which prompted the patient to turn to alcohol as a coping technique. The report describes the patient's clinical presentation, evaluation, and treatment strategy, which addressed the patient's alcohol and gambling use disorders by combining psychotherapy and pharmaceutical therapies.

## Case presentation

In March 2024, a 30-year-old married male, an auto driver by profession, belonging to the lower socioeconomic class, presented to the psychiatry outpatient department with his wife. He had complained of alcohol consumption over the past eight years, which had worsened in the last three months. This escalation was concurrent with a substantial amount of financial debt that had accrued as a result of participation in online gambling since 2018. The patient had a paternal history of alcohol dependence syndrome, while there were no known medical comorbidities nor personal or family history of psychiatric illness or self-harm.

The patient has been employed as an auto driver for the past decade, supporting his wife and three-year-old daughter. His work schedule was inconsistent, frequently requiring extended hours and late nights. The patient's family relationships and occupational functioning have been significantly influenced by his gambling addiction and alcohol use. He misses work frequently, which leads to decreased income. He also started neglecting household responsibilities and childcare duties, thereby causing strain in his marriage and comprising his daughter's well-being.

The patient's alcohol consumption started in 2015, initially during social gatherings with their peers. He consumed alcohol an average of once a week for the first five years (2015-2020), with an average of six units consumed per occasion. From 2020 to 2022, his consumption increased to three times per week, with similar quantities. His alcohol consumption has increased to 12 units per day over the past year (August 2022 to December 2023). He engaged in binge drinking for the past three months (January 2024 to March 2024), consuming up to 24 units of alcohol on three to four occasions per week. This increased consumption was largely attributed to mounting financial pressures and emotional distress from gambling losses.

In 2018, three years after initiating alcohol use, the patient was exposed to an internet-based card game called online rummy through his peers. At first, he played for one to two hours each day, spending Rs. 1,000-2,000 per game. He was strongly motivated to continue playing as a result of the substantial gains of approximately seven lakhs that he experienced from 2018 to early 2019. Nevertheless, he began to incur losses from mid-2019 to 2020. He increased his bets to Rs. 5,000-10,000 per game in order to mitigate these losses. However, his savings were completely depleted, resulting in a loss of approximately 10-15 lakh rupees.

The patient's personal and professional life was significantly disrupted by his gambling addiction. His desperate efforts to support his addiction resulted in the misappropriation of household funds and the pawning of his daughter's jewelry. Family interventions, such as confiscating his phone, were ineffective, as he would forcefully reclaim it to resume gambling. The patient was unable to resist the compulsion to gamble despite the profound guilt and shame he felt as a result of his actions and financial losses. His psychological distress and the further deterioration of his family relationships were significantly influenced by the internal struggle between the irresistible urge to continue gambling and the realization of the negative consequences. His wife reported feeling betrayed and unable to trust him as a result of his repeated fabrications regarding the misuse of family funds and gambling. The patient's relationship with his daughter was also impacted, as he spent less time at home and even sold her jewelry to support his gambling habit. The nuclear family unit experienced an increase in isolation as a result of the disappointment and frustration expressed by extended family members regarding his behavior.

The patient was admitted to the psychiatry department during his visit in March 2024 as a result of withdrawal symptoms, which included tremors, vomiting, and decreased sleep, as well as excessive alcohol consumption. The tremors occurred on a daily basis and were most severe in the mornings and interfered with daily activities for the past month. The tremors were reduced after alcohol consumption. The patient also had vomiting two to three times daily, and he reduced sleep to three to four hours per night. A physical and systemic examination upon admission revealed no abnormalities.

Mental state evaluation showed the patient to be moderately kempt with established rapport. During the interview, he exhibited increased psychomotor activity, which was characterized by restlessness, fidgeting, pacing, and an inability to remain seated. His speech was relevant and coherent, and he admitted that he struggled to resist the urge to continue gaming and felt guilty about his gambling habits. His hematological and biochemical profiles were within normal range, and thyroid function was normal. The patient was assessed using a variety of standardized assessment tools, including the Alcohol Use Disorders Identification Test (AUDIT) [7] with a score of 31, which indicates alcohol dependence syndrome; the Clinical Institute Withdrawal Assessment - Alcohol (CIWA-Ar) [8] with a score of eight, which indicates moderate withdrawal symptoms; the Beck Depression Inventory (BDI) [9] with a score of six, which indicates no clinical depression; and the Yale-Brown Obsessive Compulsive Scale (Y-BOCS) [10] with a score of six, which indicates subclinical symptoms.

Based on the DSM-5 criteria, the patient was diagnosed with gambling disorder, severe (F63.0), and alcohol use disorder, severe (F10.20) [1]. Pharmacological and psychotherapeutic interventions were implemented as part of the treatment regimen. The pharmacotherapy regimen included T. Bupropion 150 mg daily to alleviate cravings for both alcohol and gambling, T. Naltrexone 50 mg daily to reduce alcohol cravings and the rewarding effects of alcohol, T. Clonazepam 0.5 mg daily (short term) to manage sleep disturbances and anxiety symptoms, and T. Thiamine 100 mg twice daily. Due to its dual action in reducing both alcohol and gambling cravings, as well as its lower risk of side effects in comparison to other options, bupropion was administered during the initial visit.

Cognitive behavioral therapy (CBT) was implemented as part of the psychotherapy regimen, with sessions scheduled twice weekly for 45 minutes each. The primary objective was to identify and alter dysfunctional thoughts and behaviors that were associated with alcohol and gambling. Motivational enhancement therapy was also implemented to enhance the motivation to undergo change and to devise a strategy to stop alcohol use and gambling. The patient did not meet the full criteria for obsessive-compulsive disorder despite exhibiting subclinical symptoms on the Y-BOCS. Although the gambling behavior exhibited some similarities to compulsions, the diagnosis of gambling disorder provided a more comprehensive explanation. The patient underwent an eight-week inpatient treatment program. He showed a gradual reduction in both alcohol withdrawal symptoms and gambling urges during this time. The patient achieved complete remission from both alcohol use and gambling at the conclusion of the eight-week period. He demonstrated a strong commitment to maintaining abstinence and adhering to the treatment plan.

The patient was discharged with instructions to continue Bupropion and Naltrexone for an additional six months, with follow-up appointments scheduled every two weeks. The most recent follow-up was conducted three months after discharge, which was five months after the initial presentation. The patient reported that they were still abstaining from both alcohol and gambling at this time. He had resumed his employment as an auto driver and reported that his family relationships had improved. He was advised to continue attending support group meetings and follow up with the outpatient department monthly for the next six months.

## Discussion

PG, which is now referred to as gambling disorder in the DSM-5-TR, is defined as persistent and recurrent problematic gambling behavior that results in clinically significant impairment or distress. Among the diagnostic criteria are a series of behaviors, including the need to gamble with increasing amounts of money, restlessness or irritability when attempting to reduce, repeated unsuccessful attempts to control gambling, preoccupation with gambling, gambling when feeling distressed, chasing losses, lying to conceal gambling involvement, jeopardizing significant relationships or opportunities due to gambling, and relying on others for financial bailouts. The diagnosis of severe gambling disorder was warranted by the fact that our patient met all nine criteria.

PG is associated with several risk factors, including male gender, being single, divorced, or separated, experiencing stressful life events, having emotional, physical, or psychological health problems, and poor financial stability [11,12].

Individuals with a gambling disorder frequently misuse alcohol, tobacco, or other substances and may have mood or personality disorders, such as schizophrenia or antisocial personality disorder, or attention deficit hyperactivity disorder (ADHD) [13]. A systematic review and meta-analysis revealed that 57.5% of individuals who encountered negative consequences from gambling also experienced concurrent negative consequences from substance use [14]. 

Unfavorable factors in our case included male gender, comorbid substance use, heavy financial debt, and difficult interpersonal relationships. The patient's relationships were severely strained as a result of frequent lies about his gambling activities and financial losses, which resulted in a loss of trust. The case's complexity was exacerbated by the social isolation from extended family and the challenging family dynamics. The prescribed pharmacotherapy was justified by its ability to address both alcohol use disorders and gambling disorders. Bupropion was selected due to its dual action as a norepinephrine-dopamine reuptake inhibitor, which reduces both alcohol and gambling cravings. Naltrexone was prescribed to reduce alcohol cravings and prevent the rewarding effects of alcohol. The acute withdrawal symptoms, sleep disturbances, and anxiety associated with both disorders were managed through the short-term use of Clonazepam. Thiamine supplementation was essential for the prevention of Wernicke-Korsakoff syndrome in alcohol use disorder.

CBT and motivational enhancement therapy comprised the psychotherapy approach. CBT was selected due to its demonstrated efficacy in the treatment of both alcohol and gambling disorders, which enables patients to recognize and alter dysfunctional thoughts and behaviors. In order to fortify the patient's motivation for change and establish an achievable strategy for reducing alcohol and gambling consumption, motivational enhancement therapy was implemented.

Research has demonstrated the effectiveness of combined psychotherapeutic and pharmacological interventions in the treatment of co-occurring gambling and alcohol use disorders. Kraus et al. (2021) discovered that the combination of CBT and Naltrexone led to substantial decreases in both gambling and drinking behaviors [15]. Nevertheless, the relapsing nature of this disease suggests the necessity of consistent follow-up and close monitoring. Within the initial year following treatment, relapse is experienced by up to 75% of individuals with gambling disorder, according to a study conducted by Hodgins and el-Guebaly (2015) [16]. The development of integrated treatment protocols for comorbid gambling and alcohol use disorders, the long-term efficacy of combined pharmacological and psychotherapeutic approaches, the exploration of novel interventions such as neuromodulation techniques, the identification of predictors of treatment response to tailor interventions accordingly, and the development of strategies for relapse prevention and long-term management should be the primary focus of future research in this field.

## Conclusions

This case emphasizes the intricate relationship between alcohol use disorders and gambling, as well as the potential for effective treatment through an integrated approach. However, the necessity of ongoing research into effective long-term management strategies is underscored by the chronic and relapsing nature of these disorders. The extensive accessibility of online gambling platforms and the hidden promotion linked to it have resulted in increased PG disorder. The digitalization and convenience of money transactions through online websites and mobile applications have led to increased participation and awareness about online gambling. The government and society must recognize this problem and implement appropriate measures to limit the expansion of this sector.
